# Correction: Lee et al. Discrepancies Between the Tennessee Nomogram and Oncotype DX: Implications for the Korean Breast Cancer Population—The BRAIN Study. *Cancers* 2025, *17*, 3083

**DOI:** 10.3390/cancers18010119

**Published:** 2025-12-30

**Authors:** Suk Jun Lee, Joo Heung Kim, Jee Hyun Ahn, So Hyeon Gwon, Ilkyun Lee, Seho Park, Nak-Hoon Son

**Affiliations:** 1 Department of Surgery, Catholic Kwandong University College of Medicine, International St. Mary’s Hospital, Incheon 22711, Republic of Korea; atsukjun@hanmail.net (S.J.L.); iklee@ish.ac.kr (I.L.); 2 Department of Surgery, Yongin Severance Hospital, Yonsei University College of Medicine, Yongin 16995, Republic of Korea; pianac@yuhs.ac; 3 Division of Breast Surgery, Department of Surgery, Yonsei University College of Medicine, Seoul 03722, Republic of Korea; jhahn35@yuhs.ac; 4 Department of Statistics, Keimyung University, Daegu 42601, Republic of Korea; gwonsohyeon@kmu.kr

## Authorship

In the original publication, the annotation for equal authorship was not included due to editorial error [1]. The authors state that the scientific conclusions are unaffected. This correction was approved by the Academic Editor. The original publication has also been updated.
